# Activation and inhibition of *tph2* serotonergic neurons operate in tandem to influence larval zebrafish preference for light over darkness

**DOI:** 10.1038/srep20788

**Published:** 2016-02-12

**Authors:** Ruey-Kuang Cheng, Seetha Krishnan, Suresh Jesuthasan

**Affiliations:** 1 Neural Circuitry and Behavior Laboratory, Institute of Molecular and Cell Biology, Singapore; 2 NUS Graduate School for Integrative Sciences and Engineering, National University of Singapore, Singapore; 3 Neuroscience and Behavioral Disorders Program, Duke-NUS Graduate Medical School, Singapore; 4 Department of Physiology, National University of Singapore, Singapore

## Abstract

Serotonergic neurons have been implicated in a broad range of processes, but the principles underlying their effects remain a puzzle. Here, we ask how these neurons influence the tendency of larval zebrafish to swim in the light and avoid regions of darkness. Pharmacological inhibition of serotonin synthesis reduces dark avoidance, indicating an involvement of this neuromodulator. Calcium imaging of *tph2*-expressing cells demonstrates that a rostral subset of dorsal raphe serotonergic neurons fire continuously while the animal is in darkness, but are inhibited in the light. Optogenetic manipulation of *tph2* neurons by channelrhodopsin or halorhodopsin expression modifies preference, confirming a role for these neurons. In particular, these results suggest that fish prefer swimming in conditions that elicits lower activity in *tph2* serotonergic neurons in the rostral raphe.

Animals have an innate motivation to explore their surroundings, as this can help secure essential resources such as food. The pattern of exploration is shaped by characteristics of the environment, with one important feature being illumination. Animals as diverse as rodents^1^,^2^, adult fish^3^,^4^,^5^, fruit fly larvae^6^ and nematodes^7^ show a strong tendency to avoid regions that are brightly lit. Others, such as larval zebrafish^8^ and adult fruit flies^9^, prefer the light. How are these responses generated? One possibility is that light causes reflexive motor activity. In larval zebrafish, a decrease in illumination triggers turning^10^,^11^, while an increase in illumination causes forward swimming^12^,^13^. Such responses, together with other basic rules, can fully account for the ability of larval zebrafish to avoid regions of darkness^12^,^13^. However, light (or darkness for some animals) is not neutral but is innately aversive. This is suggested by observations that confining adult fish to a white compartment causes freezing^14^, and that rodents will learn an instrumental response to terminate exposure to light^15^. Moreover, anxiety, which increases the perception of threat^16^, affects preference in the light/dark assay. Drugs that reduce anxiety, such as diazepam, increase entry into regions that are normally avoided^8^,^17^,^18^, while anxiogenics increase avoidance. Thus, patterns of exploration in environments with uneven illumination reflect avoidance of an aversive stimulus.

The preference for light or darkness is further modified by factors such as time of day, level of arousal, age and even olfactory stimulation^2^,^19^,^20^,^21^,^22^, indicating that neural circuits driving the response are subject to considerable modulation^23^. One modulator that has been implicated is serotonin. Evidence for this is provided by observations that buspirone, a partial agonist of the 5HT_1A_ receptor, decreases avoidance of the aversive zone in the light-dark assay^8^,^22^,^24^, as does serotonin depletion with para-chlorophenylalanine (pCPA)^25^. In vertebrates, serotonin is produced by discrete clusters of cells, most prominently in the midbrain raphe^26^. A long-standing theory is that serotonin is released from the raphe in response to aversive stimuli^27^,^28^. Because buspirone can inhibit raphe serotonergic neurons, where 5HT_1A_ is a somatic autoreceptor^29^, the reduction of preference following buspirone treatment indicates that excitation of raphe serotonergic neurons drives avoidance. In the case of larval zebrafish, this means that darkness should cause an increase in serotonin release. However, increases in serotonin appears to be associated with increased forward swimming in the light, rather than turning away from the dark^12^. It has also been suggested that activation of the dorsal raphe encodes positive reward, rather than aversive stimuli, based on optogenetic stimulation experiments in mice^30^. Given these observations and the findings that pharmacological manipulations of serotonin signalling give conflicting results^2^,^25^ – e.g. an antagonist and agonist of 5HT_1A_ both have the same effect in the light/dark assay^25^- the role of serotonergic neurons in controlling the preference for light versus darkness is unclear.

One approach to resolve how serotonergic neurons function in a particular behaviour is to record their activity while the animal is engaged in that behaviour, or, if this is not possible, then while the animal is exposed to sensory stimuli triggering that behaviour. In mammals, this has been done to a large extent with electrical recordings^31^, and responses to both aversive and rewarding stimuli were found^32^,^33^. However, in almost all cases, the identity of the recorded cells had not been independently characterized^34^. This raises the possibility of errors in interpretation, as the raphe contains a mixture of cell types^35^. Recently, by recording molecularly-defined cells in awake mice, the dorsal raphe nucleus was found to contain serotonergic neurons with diverse responses^36^. Phasic excitation was detected, in different cells, at the presentation of both appetitive and aversive cues. Tonic firing, which appears to reflect mood or brain state^37^, was also detected. Here, again, there was variability, with some neurons firing tonically when the animal has been repeatedly exposed to aversive stimuli while others fired tonically following repeated rewards. These observations emphasize the complexity of serotonergic neurons in vertebrates, even within a single nucleus.

Larval zebrafish have a serotonergic system that is simple and yet similar to other vertebrates in many respects^38^. A major advantage of the zebrafish larva as an experimental system is its amenability to optical recording of genetically defined neurons. Hence, the response of whole populations can be analysed. Here, by combining imaging of larval zebrafish with optogenetic manipulation, we provide evidence that dark-evoked excitation operates in conjunction with light-evoked inhibition of serotonergic neurons to influence the response to light and darkness.

## Results

### Pharmacological manipulation of serotonin synthesis affects preference

**Figure 1:**
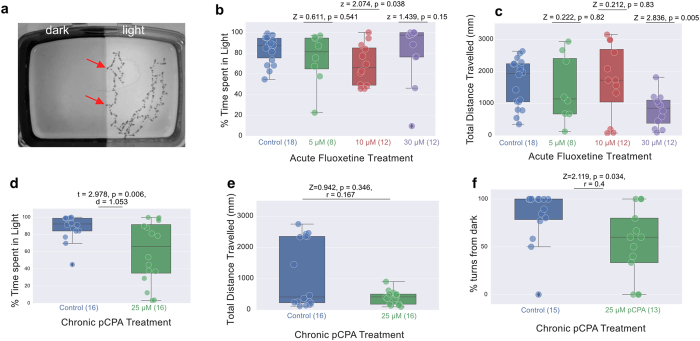
Effect of pharmacological manipulation on light preference. (**a**) An example of larval zebrafish behaviour when given a choice between light and dark zones in a tank. The image shows the trace of 1 fish, over a period of 66.7 seconds. Traces were obtained by minimum projection of a t-stack consisting of recordings made at 12 frames/second under infrared incident illumination. The fish remained within the light zone. When it approached the border (arrows), it turned back into the light. (**b, c**) The effect of three different concentrations of fluoxetine on light preference, as shown by percentage of time spent in the light (**b**) and distance travelled (**c**). The highest concentration used caused a reduction in locomotion, but did not reduce preference for the light. (**d,e**) pCPA caused a reduction in percentage of time spent in the light (**d**) and in distance travelled (**e**). Fish that approached the dark turned away less frequently following exposure to pCPA (**f**). Fish that never approached the boundary between light and dark were excluded from turn frequency analysis. In panels b-f, box plots are drawn such that the box extends between the lower (25%) and upper (75%) quartiles of the data with a line at the median. The whiskers represent the distribution of the data. Each circle represents data from individual fish and circle with black diamonds show the outliers. For distributions of data not satisfying the normality test, p-value was obtained using the Wilcoxon Rank Sum Test (Z is the test statistic, r is the non parametric effect size, panels **b,c,e,f**) else an independent T-test (t is the t-statistic, d is Cohen’s d, panel **d**) was used. Statistical comparisons were always made between control fish and drug treated fish. Number of fish in each condition is provided within brackets on the x-axis.

Larval zebrafish display a preference for swimming in light over darkness (Fig. 1a). An involvement of serotonin in larval dark avoidance has been previously suggested, based on the effects of buspirone^8^. As is the case in adults^24^, acute treatment with the selective serotonin reuptake inhibitor, fluoxetine, did not reduce preference in larvae (Kruskal-Wallis ANOVA test, p = 0.207, Fig. 1b) although the highest concentration (30 μM) significantly reduced the total distance traveled (Wilcoxon Rank Sum test, p = 0.005, Fig. 1c). Treatment with pCPA, which inhibits serotonin synthesis, increased the time spent in the dark compartment (independent t-test, p = 0.006, Fig. 1d); this is again similar to the effect seen in adult zebrafish^25^. Although there was no significant decrease in total distance traveled (Wilcoxon Rank Sum test, p = 0.346, Fig. 1e), a post-hoc visual analysis of swimming trajectory from video files indicated that pCPA-treated fish failed to turn away when they swim from the light towards darkness, in contrast to controls (Wilcoxon Rank Sum test, p = 0.034, Fig. 1f). Hence, a reduction of serotonin appears to reduce dark avoidance.

### A subpopulation of *tph2* neurons in the dorsal raphe are inhibited by light and excited by darkness

**Figure 2:**
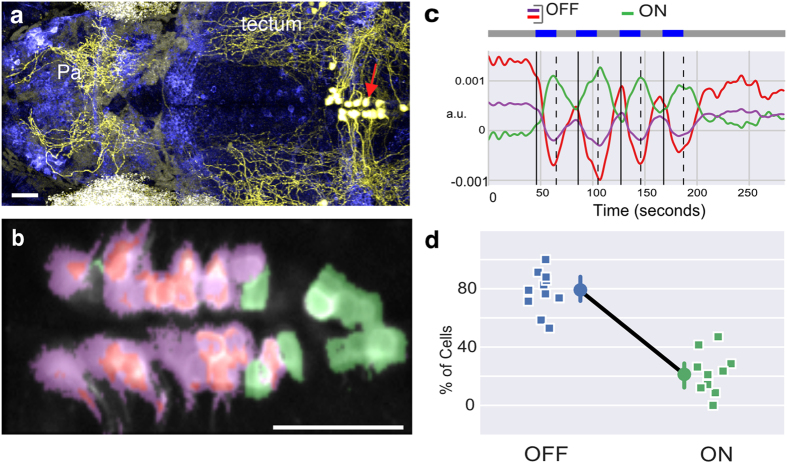
Response of dorsal raphe serotonergic neurons to light and darkness. (**a**) Serotonergic neurons (red arrow) in the dorsal raphe of a 5 day-old zebrafish, as labelled by Kaede (coloured yellow) in a Tg(*tph2:GAL4, UAS:Kaede*) line. Axons extend broadly in the brain, which is pan-neurally labelled (blue). (**b**) Activity in the raphe of a 9 day-old Tg(*tph2:GCaMP6s*) fish, in response to four pulses of light. Pixels have the same color scheme as the activity profiles shown in panel **c** (see Methods). (**c**) Time series of three clusters obtained by running k-means clustering following principal component analysis (PCA) with two components on the data in panel (**b**). Blue light was delivered during the periods indicated by the blue bars. (**d**) Average percentage of *tph2* neurons in the raphe excited (ON neurons) or inhibited (OFF neurons) by light (n = 11 fish; squares are individual fish, circles indicate mean and error bar indicates 95% c.i. around the mean). Anterior is to the left in panels (**a**,**b)** Pa: pallium; scale bar = 25 μm.

To directly assess whether serotonergic neurons in the dorsal raphe nucleus are affected by light and darkness, two-photon imaging was performed on transgenic zebrafish larvae in which the tryptophan hydroxylase 2 (*tph2*) promoter^39^ (Fig. 2a) was used to drive the genetically encoded calcium indicator GCaMP6s^40^. In the first experiment, fish were exposed to alternating periods of light and darkness (n = 11 fish, 244 cells). The majority of neurons (189 cells) displayed a drop in fluorescence during light ON (Fig. 2b–d), relative to the period of light OFF; these are termed the OFF neurons. The decrease in fluorescence persisted throughout the period of light, and was seen in all fish. A minority of cells (55 cells) displayed the opposite change, and these were located at the caudal end of the nucleus; these are the ON neurons. *Tph2* is also expressed in the pretectum^41^,^42^, and these neurons showed both ON and OFF responses (Figure S1). Hence, either light or darkness can excite *tph2* serotonergic neurons.

### Shock-induced excitation in the raphe is inhibited by light

**Figure 3:**
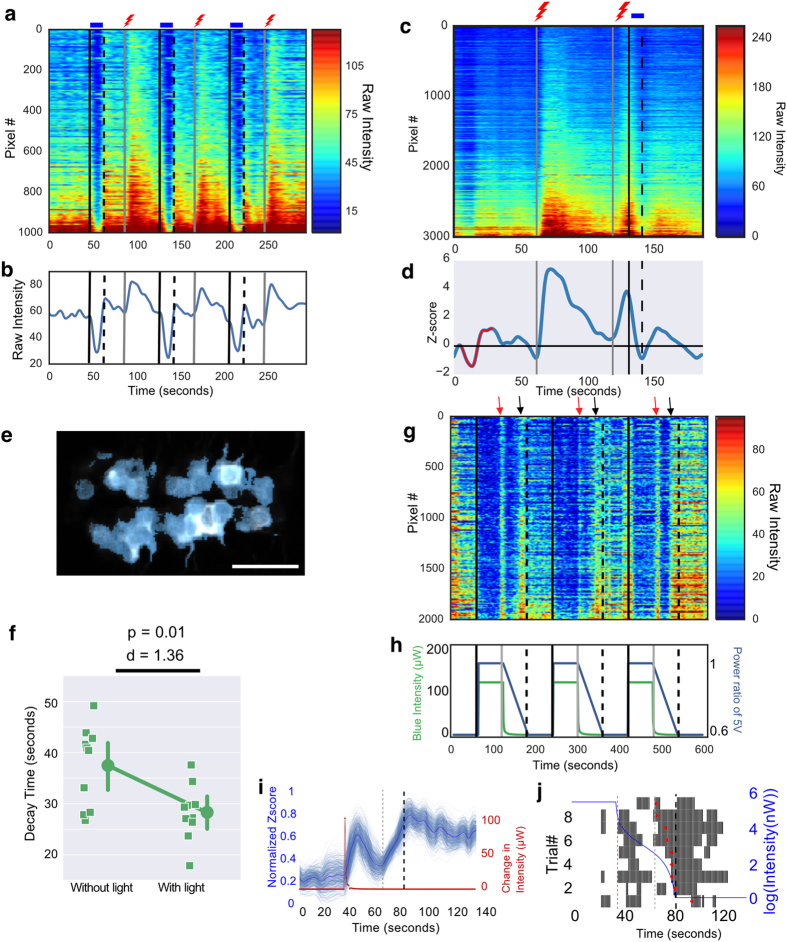
Response of dorsal raphe serotonergic neurons to shock and light. (**a,b**) Pixel-wise (**a**) and averaged (**b**) response in one fish. Light caused a decrease in activity, whereas darkness and shock caused an increase. (**c–f**) Effect of a 10-second light pulse on shock-evoked activity. Two 1-second pulses of shock were given 60 seconds apart and light was delivered 10 seconds after the second shock. (**c**) The response in one fish. (**d**) A single activity related cluster obtained from k-means clustering of the normalized data. (**e**) Cells in the raphe corresponding to the cluster shown in (**d**). (**f**) Time for shock-evoked fluorescence to drop to the minimum value, with or without delivery of light, in 10 fish. Circles represent the mean. Error bars are 95% c.i. (**g–j**) Response to gradually decreasing light. (**g**) Neurons are inhibited in the light, and excited at the start of the decrease (red arrows), and close to darkness (black arrows). (**h**) Voltage applied to the LED (blue trace) and measured intensity (green trace). **(i)** Data compiled from three fish, each exposed to three trials (total of 9 trials). The red trace shows difference in intensity between two consecutive time points. The bold blue trace is the mean activity across 9 trials. The thinner lines represent resamples of the data obtained by 500 bootstrap iterations. There is a transient excitation following the large change in illumination. (**j**) Raster plots showing spike times obtained by temporal deconvolution of the mean calcium traces in each trial. The trials are sorted in ascending order of the half maxima. Black lines correspond to individual spikes of action potentials. The red diamonds indicate the half maxima of the second peak response to decreasing illumination. In 8/9 trials, the second response starts before the LED intensity turns 0. Red dashed line: time point with the large change in illumination. Grey dashed line: earliest half maxima. Bold black dashed line: time-point when the LED intensity was 0. p-value in (**f**) is based on Paired T-test. d is Cohen’s d. Scale bar = 25 μm.

We tested how raphe neurons that are inhibited by light and excited by darkness (the OFF neurons) respond to a different stimulus, to determine whether excitation is specific to darkness. To do this, fish were exposed to a transient electric shock as well as alternating periods of light and dark. OFF neurons were excited by the shock (Fig. 3a,b; n = 13 fish; 308 cells), with shock-induced excitation lasting beyond the duration of the shock. A similar excitation was also seen in the caudal raphe, indicating that the excitation here may not be tied strictly to reward value, because both shock (an aversive stimulus) and light (which is not aversive) triggered excitation.

The sustained excitation in dark-excited raphe neurons appeared to be inhibited by the delivery of blue light. This was confirmed by comparing the time for GCaMP6s fluorescence to reach a minimum after the shock, with or without the presence of light (Fig. 3c–f; n = 10 fish, 253 cells; Cohen’s d = 1.36, Paired T-test p = 0.01). Hence, an increase in irradiance can reduce tonic activity of serotonergic neurons following an aversive stimulus of a different modality. This indicates that the effect of light on raphe neurons is not simply a loss of dark-induced excitation, but is an active inhibition.

We next imaged fish that were exposed to a gradual decrease in illumination, by progressively reducing the voltage supply to the LEDs. This was primarily an attempt to mimic illumination changes that would accompany movement of the fish towards darkness in a light/dark assay. Two discrete excitation responses were detected, one correlating with the start of decrease in intensity, and the second towards the end (Fig. 3g; n = 3 fish, 66 cells). The presence of two responses may be caused by the fact that decrease in light intensity was unexpectedly non-linear in our experiment (Fig. 3h). This pattern of change indicates that a sharp change in luminance (decrease of 107 μW) as well as the absence of light below a threshold (average luminance of 0.107 ± 0.11 μW), which are both aversive stimuli^43^, can cause excitation in a population of dorsal raphe serotonergic neurons (Fig. 3i,j). Excitation to the sharp drop in luminance was transient (average duration of response: 10.22 ± 4.68 s, n = 3 fish), in contrast to the sustained excitation seen with shock (average duration of response: 30.09 ± 0.75 s, n = 10 fish).

### Optogenetic manipulation of tph2 serotonergic neurons alters preference

**Figure 4:**
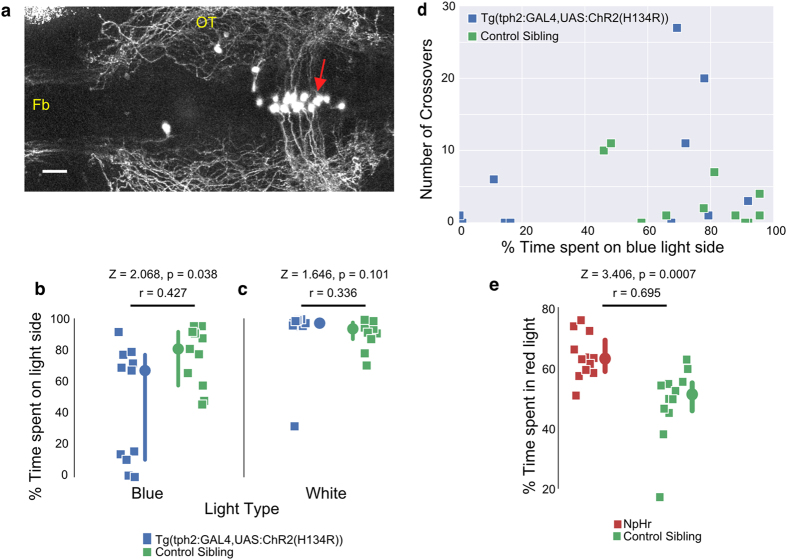
Effect of optogenetic manipulation of *tph2* neurons on preference. (**a**) Dorsal view of a *Tg(tph2:GAL4, UAS:ChR2(H124R)-eYFP)* larva, with anterior to the left. Channelrhodopsin is expressed in the dorsal raphe (red arrow). The image is a maximal projection of optical sections covering 122 μm. (**b,c**) The percentage of time spent in the light side of the tank, with either blue (**b**) or white (**c**) light. All control sibling fish preferred the side with blue or white light. However, fish expressing ChR2 (H134R)-eYFP under the *tph2* driver had a significant preference for the dark side when blue light was used. (**d**) The number of crossovers between the blue light and the dark side as a function of their percentage of time spent on the blue light side. (**e**) Preference of eNpHR3.0-expressing fish and their siblings for red over green (n = 12 each). There is a significant change towards red in fish expressing halorhodopsin (p = 0.0007). Fb: forebrain; OT: optic tectum; scale bar = 25 μm. Gamma = 0.65 was applied to panel (**a**). In panels c and e, each square represents one fish (n = 11 fish in each group). Circles are the median and error bar indicates 95% c.i. around the median. p-value is obtained from Wilcoxon Rank-sum test. Z is the test statistic; r is the non-parametric effect size.

If avoidance of the dark is caused by an increase in the activity of raphe serotonergic neurons in darkness, then triggering activity in the light should induce avoidance of light. To test this, we expressed a variant of channelrhodopsin, ChR2 (H134R)^44^, using the *tph2* driver. The presence of an eYFP tag enabled identification of fish and neurons carrying the transgene (Fig. 4a). Fish were placed in a chamber with a choice between blue light (20 W/mm^2^) and darkness. While some fish expressing ChR2 (H134R)-eYFP retained a preference for the blue light, a significant number displayed a preference for the dark (Fig. 4b). After the blue light/dark assay, all fish were immediately transferred to a normal white light/dark assay as a control and all fish stayed in the light (Fig. 4c). An analysis of the number of crossovers in the blue light experiment (Fig. 4d) indicates that there are three classes of behaviour – fish that stay in the dark, those that stay in the light and those that cross several times. Siblings not expressing ChR2 (H134R)-eYFP, in contrast, remained in the light and rarely crossed over.

The bimodal response seen with ChR2, i.e. the preference for either light or darkness, could be due to several reasons (see Discussion). One possibility is that excitation of a subset of serotonergic neurons is rewarding, while excitation of another subset signals punishment, with random expression driving greater activation in one of the subsets. In this scenario, inhibition of the first subset would be non-rewarding, while inhibition of the second is rewarding. Hence, optogenetic inhibition of *tph2* neurons in a two-choice assay would be predicted to also give a bimodal distribution. To test this, we placed fish expressing halorhodopsin (the eNpHR3.0 variant^45^) in *tph2* neurons, in a chamber where they could swim either in green or red light, which have differential effects on halorhodopsin. Control fish, which were siblings without halorhodopsin expression, spent equal time in both regions of the tank, whereas the eNpHR3.0 expressing fish showed a shift towards the red light (Fig. 4e). This uniform shift is inconsistent with a dual role for inhibition in preference.

## Discussion

We have investigated the mechanism underlying the preference of larval zebrafish for light over darkness. Pharmacological manipulation with pCPA indicates that serotonin has a role in causing dark avoidance, consistent with the published effect of buspirone^8^. Activity imaging demonstrates that darkness excites a majority of *tph2* serotonergic neurons within the raphe, a structure that has been implicated in response to aversive stimuli^27^, further supporting the possibility that release of serotonin is involved in dark avoidance. Moreover, expression of ChR2 under the *tph2* driver, which would enable blue light to depolarize serotonergic neurons, yielded fish with reduced preference for blue light. Together, these results indicate that excitation of *tph2* serotonergic neurons influences avoidance of darkness, potentially by increasing anxiety. However, not all *tph2* serotonergic neurons are excited by darkness. Some of these neurons, which are located more caudally in the raphe, are inhibited by darkness and excited by light. The dorsal raphe of zebrafish, like those of other vertebrates, consists of two subnuclei - B6 and B7 - that are organized rostro-caudally and have distinct targets, inputs and co-transmitters^41^. The responses to light and darkness may thus be segregated by subnuclei, indicating that serotonergic neurons have more than one role in the preference for light.

Some evidence for this is provided by the bimodal response seen in fish expressing ChR2 in *tph2* neurons: while many avoid the light, a number retain dark avoidance. This is unlikely to be due simply to lack of ChR2, as expression in the raphe was confirmed in all fish. The bimodal response may reflect the fact that additional factors, such as internal state, act via other pathways to over-ride the effects of serotonin in some fish. Alternatively, where the fish swims may depend on the relative level of serotonergic neuron activation under different stimuli, with there being a winner-take-all outcome. For example, in some fish, stronger depolarization of dark-excited neurons by blue light relative to darkness, due to higher levels of ChR2 expression, may cause avoidance of the light; in others, stronger activation of these neurons by darkness may lead to avoidance of the dark.

Another possibility is that excitation in some raphe neurons is rewarding^30^,^46^,^47^. It might be argued that a stochastically higher level of expression in one subset of neurons biases preference. However, photoinhibition by expression of eNpHR3.0 with the same *tph2* driver did not lead to a bimodal outcome in a green/red choice assay. Thus, the assumption that some raphe neurons encode for reward and others encode for punishment was not supported by our data because that assumption will predict a bimodal result in both photostimulation (ChR2) and photoinhibition (eNpHR3.0) experiments.

Excitation of dorsal raphe neurons has been linked to arousal^39^. We suggest that the excitation seen in the caudal raphe, which occurs in the light, may be involved in this phenomenon. Darkness does not appear to trigger arousal, as arousal is associated with prolonged excitation of the raphe^39^, and sudden darkness only causes transient excitation (Fig. 3g–j). Moreover, extended darkness, in contrast to light, does not cause increased locomotion^11^, which is one feature of arousal. It is known that the level of aversion to darkness is dependent on the contrast between the light and dark zone^8^. Activity in dorsal raphe increases perception of a light/dark boundary during arousal^39^. Hence, the ChR2-expressing fish may have a heightened avoidance of the dark because of increased visual sensitivity. This, coupled with the aversive effect of excitation of the anterior raphe by blue light, could generate the bimodal response.

We have found that light inhibits the excitation of raphe serotonergic neurons triggered by a shock. This suggests that light can actively inhibit activity induced by an aversive stimulus, and may thus have some rewarding properties. This would be consistent with previous experiments in rodents demonstrating the rewarding effects of intra-cranial self-administration of the GABA_A_ agonist muscimol^48^ or of microinjection of the 5HT-1_A_ agonist 8-OH-DPAT^49^ directly into the dorsal raphe nucleus. A rewarding effect of light in the light/dark preference assay may not have been detected previously^12^, as reward value in this case appears to be in the form of relief from aversion; light would not trigger approach in the absence of an aversive stimulus. The inhibitory effect of light may also explain null effect of acute fluoxetine treatment, which blocks serotonin reuptake after it is released at the synapse. If the neurons controlling aversion are already inhibited by light, this compound cannot further increase the level of serotonin.

In conclusion, the data here indicate that both excitation and inhibition of *tph2* serotonergic neurons, most likely in the raphe, influence the preference of larval zebrafish for light over darkness. Potential targets of these neurons are regions of the forebrain that appear to be involved in this behaviour^22^. These data also support the picture that raphe serotonergic neurons are multifunctional^34^,^36^. We propose that the zebrafish can provide one experimental model to further investigate the principles of serotonin effects on vertebrate brain circuits, as it is a relatively simple system that enables a combination of behaviour and activity imaging of defined cells across the entire brain. To probe the role of neurons with distinct response characteristics, however, it will be necessary to use finer-grain drivers. While serotonergic neuron-specific drivers, such as the *tph2:GAL4* driver used here, are better than wholesale activation of a particular region or pharmacological manipulation of whole tissues, the complexity of response suggests that manipulation needs to be carried out at the resolution of subnuclei or response types. Intersectional strategies may be useful in this respect, and will help clarify the effects of different levels of activity in different functional groups. This will enable the formulation and testing of theories that more fully reflect the complexity of serotonin function in the vertebrate brain.

## Methods

### Zebrafish lines

Experiments were carried out in accordance with protocols approved by the Institutional Animal Care and Use Committee of Biopolis. Zebrafish (*Danio rerio*) lines used for this study were: *Tg (UAS:GCaMP6s)sq202*, *Tg (tph2:GAL4, UAS:Kaede)y228*^39^, *Tg (UAS:ChR2 (H134R)-eYFP)s1990t*^44^, *Tg (UAS:eNpHR3.0-mCherry)s1987t*^44^ and AB wildtype.

GCaMP6s^40^ (Addgene plasmid 40753) was cloned downstream of UAS in a Tol2 vector, using the Gateway system, to create *Tg(UAS:GCaMP6s)sq202*.

Imaging was carried out on larvae in a *nacre*^−/−^ background^50^. Animals were housed in a facility with lights on between 8 am and 10 pm. Larvae were grown in batches of 25–30, in 500 ml water from age 4 dpf. Animals were sorted on the basis of fluorescence for growing up, and selected at random for imaging.

### Drug treatments

#### Fluoxetine

Fluoxetine hydrochloride (Sigma-Aldrich F132) was prepared as a 5 mM stock in E3 fish water (5 mM NaCl, 0.17 mM KCl, 0.33 mM CaCl2, 0.33 mM MgSO4). Acute treatment was carried out using 3 concentrations (5, 10 and 30 μM), based on previous reports using bath application in larval zebrafish^12^,^51^. At 14 dpf, larval zebrafish were put in the test tank for 10 minutes for habituation. The drug was then pipetted into the tank for another 10 minutes of acclimatization. After that, the fish were subject to the light/dark assay for another 10 minutes to observe acute drug effects. Controls were siblings from the same batch of experimental fish, which were treated in the same timeline except that the drug treatment was omitted.

#### pCPA

The tryptophan hydroxylase inhibitor pCPA (4-Chloro-DL-phenylalanine methyl ester hydrochloride; Sigma-Aldrich C3635) was dissolved in E3 fish water to make a 10 mM stock solution. Chronic treatment began by exposing 13 dpf larval zebrafish, in a group of 8–12 fish per dish, to 25 μM pCPA^52^. The light/dark test began 24 hours later, first with 10 minutes of acclimatisation in the test tanks. Fish were maintained in E3 with 25 μM pCPA throughout the experiment. Controls, which were siblings of the drug treated fish, were exposed to E3 fish water for 24 hours and tested in E3.

### Calcium imaging

Zebrafish larvae (aged 5–11 dpf) were anaesthetized in mivacurium and embedded in low-melting temperature agarose (1.2–2.0% in E3) in a glass-bottom dish (Mat Tek). They were imaged on a Nikon two-photon microscope (A1RMP), attached to a fixed stage upright microscope, using a 25x water immersion objective (NA = 1.1). The femtosecond laser (Coherent Vision II) was tuned to 920 nm for GCaMP6s imaging. Images were collected in galvano-scanning mode with a 525/50 nm bandpass emission filter at 1 Hz. Fish were imaged during the day only, between 10 am and 7 pm. The facility lights were on from 8 am to 10 pm.

Light pulses were generated by a TMS Lite LED box with the peak wavelength at 470 nm. This was powered by a 24V adapter and controlled by TTL signals synchronized with image capture using a National Instruments DAQ board, all controlled by Nikon Elements software. A low-pass filter (450 nm “Tokyo blue 071”, Lee Filter) was used to reduce bleed-through of the light to the detector. Light intensity at the sample was 20 mW/mm^2^. To create blue light with gradually decreasing intensity, a DS1804 digital potentiometer was used to control a pair of 5 mm blue LEDs powered and controlled by an Arduino board. Light intensity was measured using the PM100A meter and S120VC sensor (Thorlabs).

The electric field stimulus was created by placing electric wires below the water surface. The wires were 4 cm apart oriented parallel to the anterior-posterior axis of the fish and connected to a Grass SD9 Stimulator providing 50 V, thus creating a 12.5 V/cm electric field. The electric field was applied for 1 second at 50 Hz.

### Data analysis

#### Image Processing

Raw images from each z-plane were first sent through an x-y registration protocol to correct for horizontal/vertical shift after the first image by using *normxcorr2* in Matlab (Mathworks). Where necessary, a median filter was applied to reduce noise and data was normalised using 25 seconds before light onset as a baseline (red trace shown in Fig. 3d). Imaging data was analysed using custom scripts in Python and plots were created using matplotlib and seaborn.

#### Data Analysis

Raw data in Fig. 3a,c,g were plotted by extracting pixels with high standard deviations corresponding to the raphe (around 1000–3000 pixels) and sorting them by their average response. To provide a spatial representation of activity, the Thunder algorithm^53^ was used. For Fig. 2b,c, PCA was performed to reduce noise and obtain a low dimensional spatial representation of pixels with similar temporal profiles. PCA components representing light evoked activity patterns that captured over 70% of the variance were used to reconstruct the data to a reduced dimension. *K*-means clustering was performed following PCA to identify signals with similar response profiles. For Fig. 3d and Figure S1 which had reduced noise, *K*-means was directly performed on the normalised data. *K*-means clustering partitions the *n* pixels into *k* clusters where the *n* pixels belong to a cluster with the closest mean. Each pixel in the raw data could then be grouped into the *k* different clusters it belongs to and differently coloured. This is shown in Figs 2b and 3e. With this, individual cells can be seen clearer and can be manually segmented/counted for response properties if necessary (for example in Fig. 2d).

Decay time in Fig. 3f was calculated by measuring the time taken for shock evoked activity to reach a global minimum over 50 seconds after shock delivery.

#### Analysing response to intensity change

To understand effects of intensity changes, fish were kept in 60 seconds of darkness before switching on the LED which remained at high intensity for 60 seconds after which the LED sharply decreased by 107 μW at 61 seconds and then gradually decreased to total darkness (0 W power) over the next 60 seconds. This is plotted in Fig. 3h. Three such trials were done on three different fish. The nine trials were then aligned to 20 second after light onset and 50 second after the LED’s power went to zero. To find if there is a threshold of light intensity below which the raphe neurons are activated in the intensity gradient experiment, mean calcium traces from each trial were converted to firing rates by temporal deconvolution^54^. Since calcium signals do not have a sharp rise and decay time, the estimated firing rates provided an easier way to calculate threshold. The firing rates were converted to spikes if they were 2 standard deviations above the mean and spike times were plotted as a raster plot in Fig. 3j. The peak firing rate after the LED went to 0W was calculated and the half maxima was defined as the time point that is 50% of the peak response.

#### Statistics

Prior to performing statistical comparisons, the Shapiro-Wilk test was used to confirm the null hypothesis that the data follows a normal distribution. If the null hypothesis was rejected, a non parametric Wilcoxon Rank Sum test was used and the median ±95% confidence interval around the median of the distributions were reported. If the distribution were normal, a Paired or independent student t-test was used and the mean ±95% confidence interval around the mean were reported. Effect sizes were reported when the sample sizes were comparable. When data was normally distributed, Cohen’s d was used. For non-normal distributions, a non parametric effect size was calculated as r = z/√N where z is the test statistic obtained from Wilcoxon Rank sum test and N is the total number of samples. For drug experiments when multiple concentrations were used (i.e. fluoxetine treatment), a one-way ANOVA on ranks (Kurskal-Wallis rank test) was used to determine treatment effect.

### Optogenetic manipulation

Previously validated lines^44^ carrying the H134R variant^55^ or eNpHR3.0 variant^45^ fused to eYFP and mCherry respectively were used. Fish were sorted with a fluorescence stereomicroscope prior to behavioural testing. To activate ChR2 (H134R)-eYFP, a LED blue light box was used with intensity measured at 20 mW/mm^2^. The light was filtered by a low-pass (450 nm) filter (Tokyo Blue 071 from LEE Filter). For the light choice experiment, green (525 nm) and red (660 nm) light boxes (TMS Lite) were used, with intensity at 50 mW/mm^2^. Expression of ChR2 (H134R) and eNpHR3.0 was confirmed by imaging for the YFP or mCherry tag after behavioural testing using two-photon microscopy. All fish had expression in the dorsal raphe; expression in other regions, such as the pretectum, was not reproducible and did not correlate with behaviour.

### Light/dark assay

The assay was conducted in transparent plastic tanks (dimensions: 43 mm width × 60 mm length × 30 mm height) filled with 50 mL of embryo water (840 mS) in a dark procedure room. To create the dark component, half of tank was covered by black 3M Vinyl tape on the exterior surface. Four such tanks were monitored simultaneously by a Logitech webcam (Model# Pro 9000) from above in a resolution of 800 × 600 pixels. To prevent social influence among fish, a black cardboard was placed between the tanks. To prevent influence from the experimenter, a cardboard box was used to isolate the entire set up such that the fish could not see the experimenter or the computer screen. To create the light compartment, white light was provided from below the tank by an Apple iPad Air in the strongest brightness level.

Real-time tracking of the fish location was achieved by custom-written Python codes using the opencv library. The recording lasted 10,000 frames with a speed of 16 fps, which equals to about 10.5 minutes. After the experiment ended, the x-y coordinates of each fish in each frame were exported to a Microsoft Excel file for further processing. When fish entered the dark side of the tank, the tracking algorithm would lose the fish. In such frames, 0-0 (x-y coordinates) was written to the computer memory. In the Excel sheet, a custom-written macro calculated the number of frames with 0-0 and the number of frames with non-zero x-y coordinates. Both numbers are divided by 10,000, thus representing the percentage of time the fish spent in the dark side and the light side.

The number of crossovers was calculated as the total number of frames where the fish swam from the light side to the dark side and stayed for at least 1 second. Frequency of turning away from the dark side was calculated by first determining the number of approaches to the light/dark boundary. This was defined as movement to within two body lengths (about 10 mm) of the boundary, in a trajectory that was close to perpendicular to the boundary. The fish was considered to turn if it changed direction by 90 degrees or more, while within the two body-length area in the light area or while within one body length into the dark side. The total number of turns divided by the total number of approaches is represented as percentage of turns from dark in Fig. 1f. A few fish were discarded from this analysis because they never moved to the boundary area during the experiment.

These automated (except the analysis of turn away from the dark side) analyses were applied equally to all fish. No animals were excluded. The sample size required was based on pilot studies using 40 wildtype fish from 4 different batches to establish the robustness of the assay, and a pilot study with ChR2 expressing fish. 3 independent batches of ChR2 fish were tested. The light choice assay was carried out on 2 independent batches of eNpHR3.0 fish, and sample size was based on a pilot study with a separate batch.

## Additional Information

**How to cite this article**: Cheng, R.-K. *et al.* Activation and inhibition of *tph2* serotonergic neurons operate in tandem to influence larval zebrafish preference for light over darkness. *Sci. Rep.* **6**, 20788; doi: 10.1038/srep20788 (2016).

## Supplementary Material

Supplementary Information
