# Supplementary material for: Activation and inhibition of tph2 serotonergic neurons operate in tandem to influence larval zebrafish preference for light over darkness
Source: Sci Rep. 2016 Feb 12;6:20788. doi: 10.1038/srep20788 (PMC4751628; doi:10.1038/srep20788)
Supplement: Supplementary Information [file srep20788-s1.pdf]

Activation and inhibition of tph2 serotonergic neurons operate in tandem to influence larval zebrafish preference for light over darkness

Ruey-Kuang Cheng[1], Seetha Krishnan[2] and Suresh Jesuthasan[1, 3, 4, *]

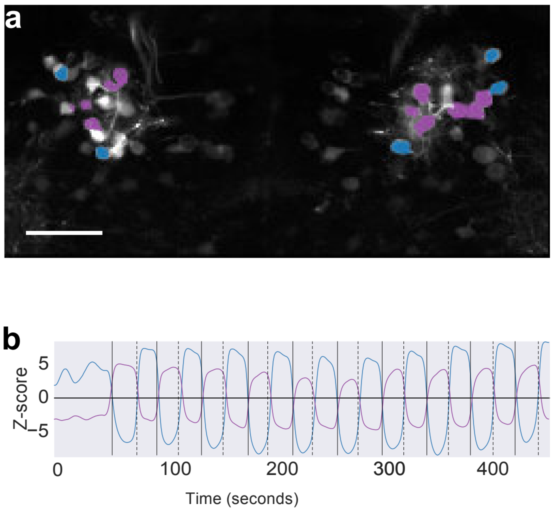

Figure S1. Response of pretectal *tph2* neurons to light and darkness. (**a**) Dorsal view of the pre-tectal area of a 11 dpf *Tg(tph2:GAL4, UAS:GCaMP6s)* fish, with anterior at the top. The purple colour indicates cells that are excited by light (ON cells), whereas the blue colour indicates cells that are excited by darkness (OFF cells). (**b**) Response of pretectal *tph2* neurons to light and darkness, determined using k-means clustering of a time-series recorded at 1 Hz. The series was analysed using the Thunder platform. Scale bar = 25 µm.
